# Supplementary figures and images for: The Dynamics of Tumor-Infiltrating Myeloid Cell Activation and the Cytokine Expression Profile in a Glioma Resection Site during the Post-Surgical Period in Mice
Source: Brain Sci. 2022 Jul 7;12(7):893. doi: 10.3390/brainsci12070893 (PMC9313002; doi:10.3390/brainsci12070893)

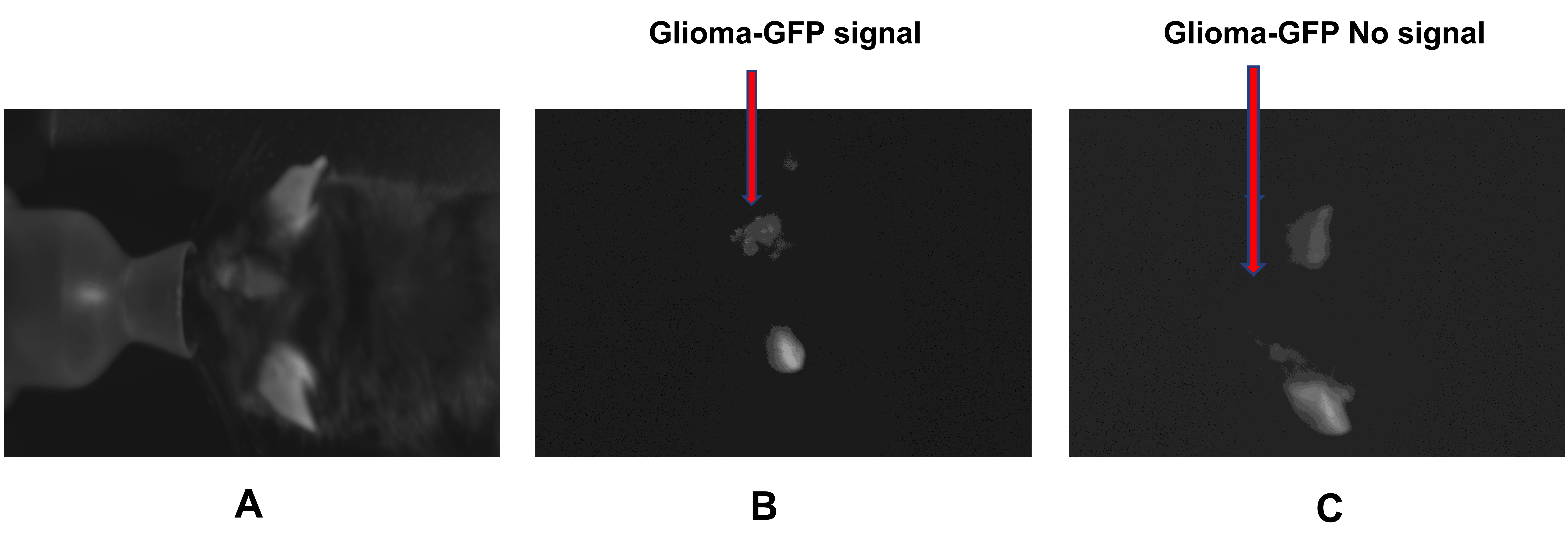

Supplement: Supplementary file 1 [file brainsci-12-00893-s001.zip › Figure S1.tiff]

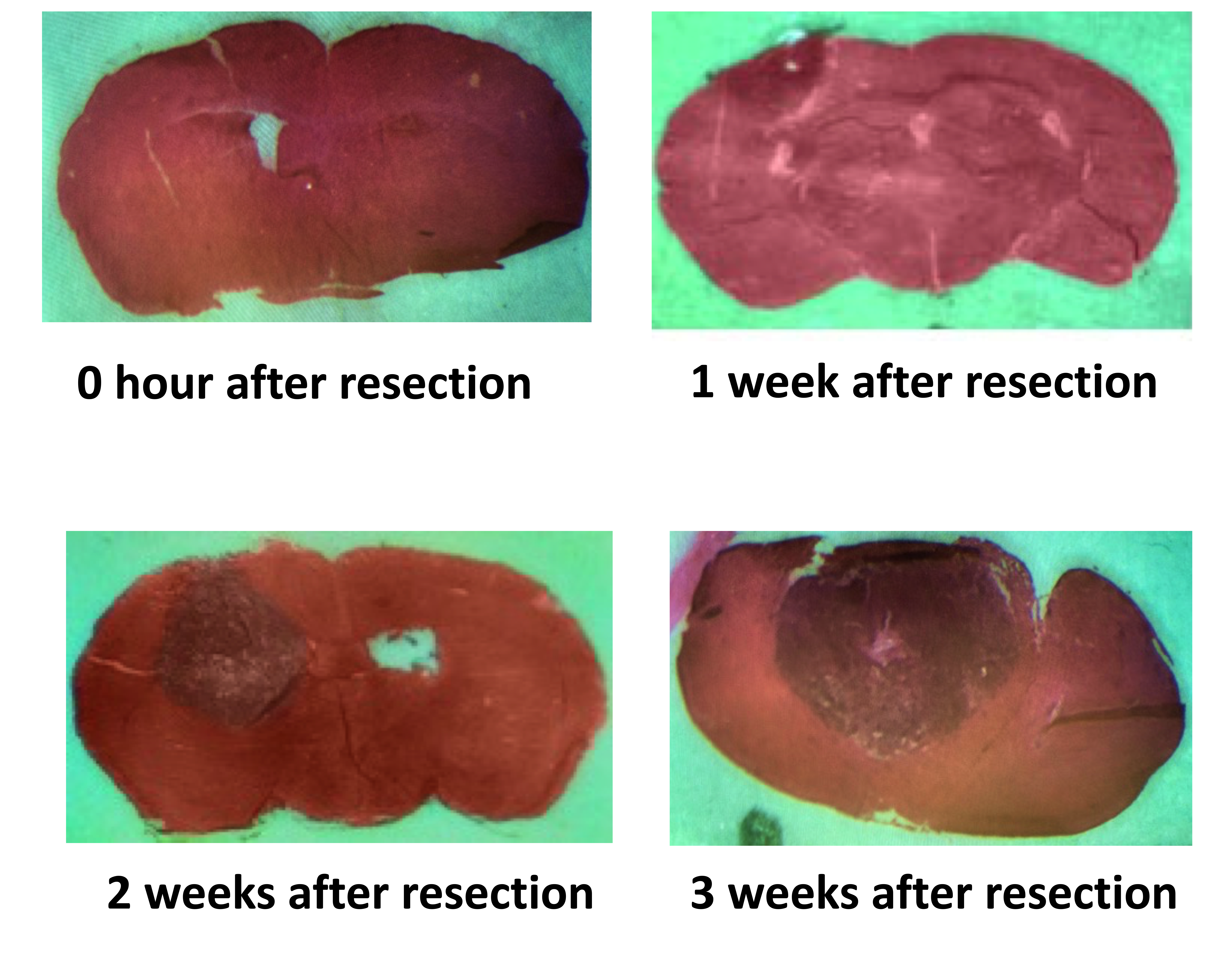

Supplement: Supplementary file 1 [file brainsci-12-00893-s001.zip › Figure S2.tiff]

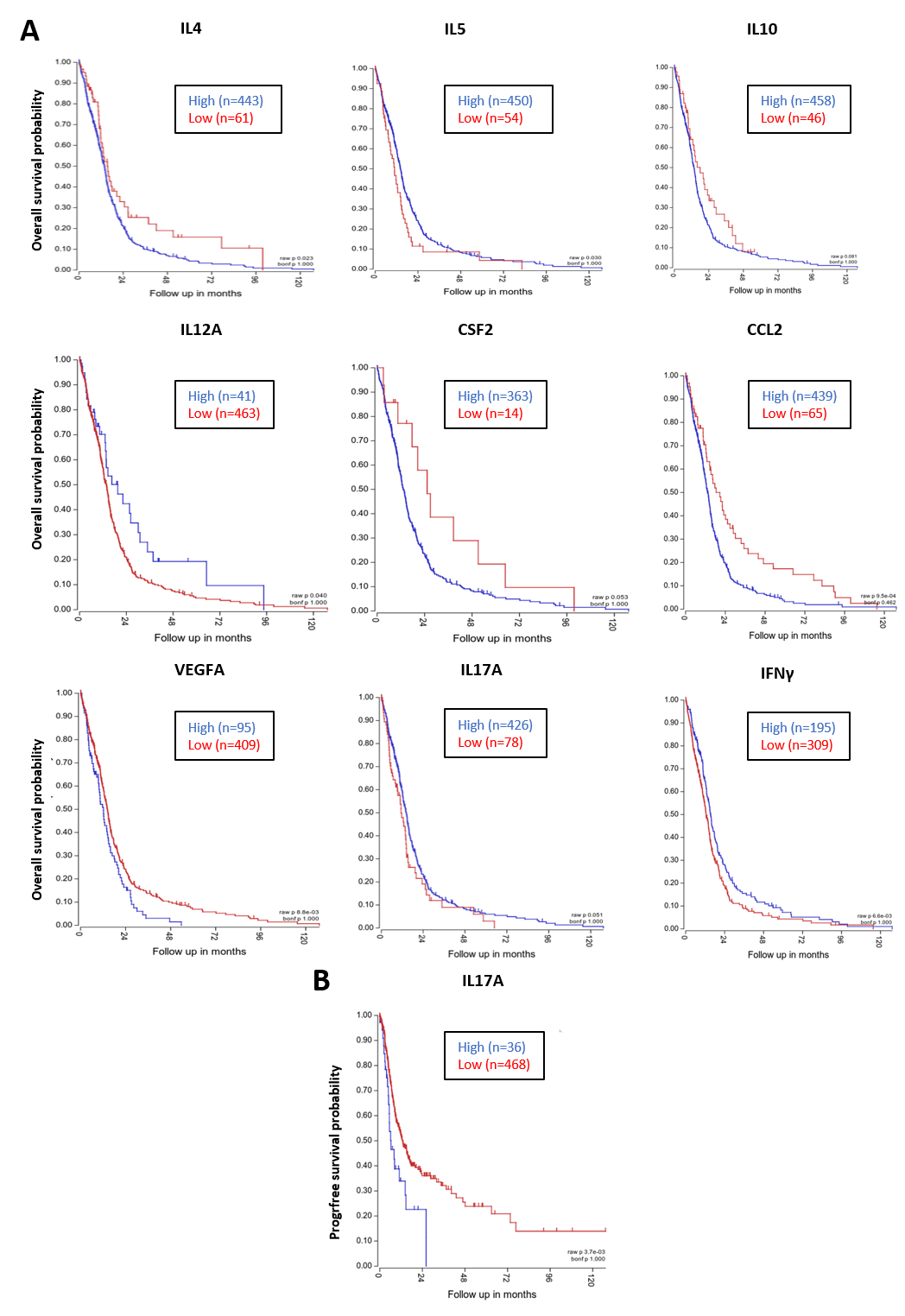

Supplement: Supplementary file 1 [file brainsci-12-00893-s001.zip › Figure S5.tif]

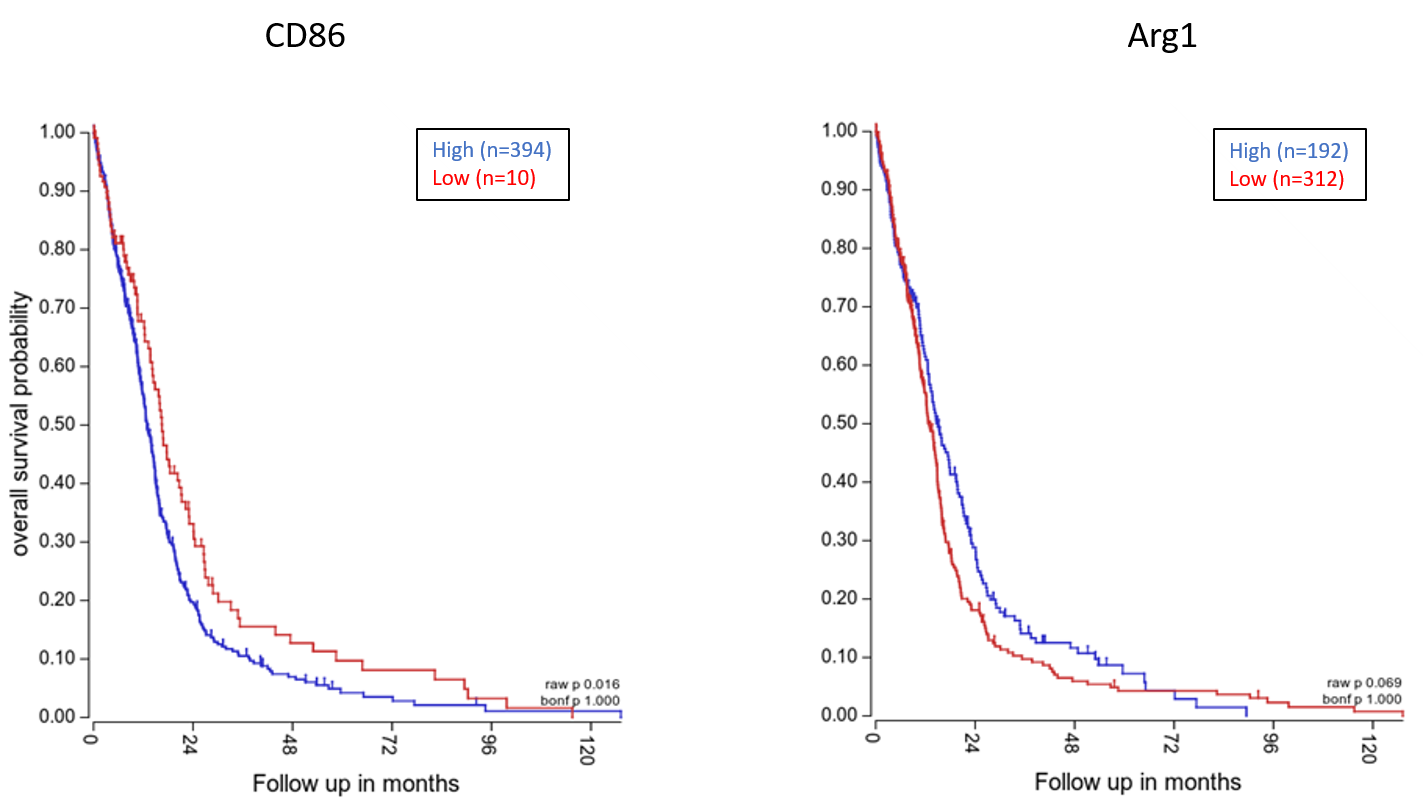

Supplement: Supplementary file 1 [file brainsci-12-00893-s001.zip › Figure S6.tif]
